# Beyond sepsis: *Staphylococcus epidermidis* is an underestimated but significant contributor to neonatal morbidity

**DOI:** 10.1080/21505594.2017.1419117

**Published:** 2018-02-27

**Authors:** Ying Dong, Christian P. Speer, Kirsten Glaser

**Affiliations:** a University Children's Hospital, University of Wuerzburg, Wuerzburg, Germany; b Department of Neonatology, Children's Hospital of Fudan University, Shanghai, China

**Keywords:** bronchopulmonary dysplasia, inflammatory disorders, innate immunity, necrotizing enterocolitis, neonatal sepsis, preterm infants, *Staphylococcus epidermidis*, white matter injury

## Abstract

*Staphylococcus epidermidis* accounts for the majority of cases of neonatal sepsis. Moreover, it has been demonstrated to be associated with neonatal morbidities, such as bronchopulmonary dysplasia (BPD), white matter injury (WMI), necrotizing enterocolitis (NEC) and retinopathy of prematurity (ROP), which affect short-term and long-term neonatal outcome. Imbalanced inflammation has been considered to be a major underlying mechanism of each entity. Conventionally regarded as a harmless commensal on human skin, *S. epidermidis* has received less attention than its more virulent relative *Staphylococcus aureus*. Particularities of neonatal innate immunity and nosocomial environmental factors, however, may contribute to the emergence of *S. epidermidis* as a significant nosocomial pathogen. Neonatal host response to *S. epidermidis* sepsis has not been fully elucidated. Evidence is emerging regarding the implication of *S. epidermidis* sepsis in the pathogenesis of neonatal inflammatory diseases. This review focuses on the interplay among *S. epidermidis*, neonatal innate immunity and inflammation-driven organ injury.

## Introduction

Steady progresses in the fields of perinatology and neonatology have improved neonatal survival, especially in very immature preterm infants [1,2]. However, the burden of neonatal morbidities, such as bronchopulmonary dysplasia (BPD), white matter injury (WMI), necrotizing enterocolitis (NEC) and retinopathy of prematurity (ROP), remains substantial [1,2]. Besides these inflammatory disorders, neonatal sepsis is ranked as one of the major morbidities in preterm neonates [1,2]. *Staphylococcus epidermidis*, the leading species of coagulase-negative staphylococci (CONS), has emerged as the predominant pathogen of sepsis in preterm infants [3]. Due to its ubiquitous colonization on human skin, *S. epidermidis* has been conventionally considered as a harmless commensal [4]. However, mortality due to *S. epidermidis* sepsis, defined as one or more positive blood cultures with corresponding clinical signs, ranges from 1.9% to 4.8% in the general neonatal population, and may be as high as 9.4% in very low birth weight (VLBW) infants [5-9]. Moreover, a growing body of evidence implicates a strong association between *S. epidermidis* sepsis and inflammation-related neonatal morbidities, such as BPD, WMI, NEC and ROP [10-16]. Although not fully delineated, in each entity, the pathogenesis is considered to be a multiple-hit process with inflammation being a principal downstream mechanism [17-20]. Prenatal and postnatal injurious events, such as chorioamnionitis, resuscitation, oxygen toxicity, mechanical ventilation and neonatal infection, have been demonstrated to contribute to dysregulated and sustained inflammation that may severely impair organ development, especially in very immature preterm infants [17-20]. Maturation-dependent characteristics and genetic propensity may underlie the individual vulnerability for extrinsic insults [17-20].

Neonatal sepsis is a major risk factor of adverse neonatal inflammation, and considerably contributes to neonatal short-term and long-term morbidity by inducing, exacerbating or perpetuating an imbalanced host inflammatory response [17-21]. *S. epidermidis* is likely to be implicated in sepsis-induced neonatal inflammation, and thus might play an important role in inflammatory-driven organ injury. As compared to other pathogens, such as *Staphylococcus aureus* and *Escherichia coli*, *S. epidermidis* may be underestimated in terms of its role in neonatal morbidity. This review summarizes data from current clinical and experimental studies to better elucidate the pathogenic mechanisms of *S. epidermidis*, host response to *S. epidermidis*, and the role of *S. epidermidis* sepsis-induced inflammation in the pathogenesis of neonatal inflammatory disorders. A thorough understanding in this area may help to optimize current anti-infectious strategies and to improve neonatal outcomes, especially in very immature preterm infants.

## Epidemiology and population structure of *S. epidermidis* in neonates

The colonization of *S. epidermidis* in humans starts immediately after birth [22]. Despite wide interpersonal variations and differences in terms of body sites and age within the same host, *S. epidermidis* remains to be the most frequently isolated species of skin microbiome [23]. Recent data indicate that *S. epidermidis* may belong to the abundant bacterial genera in airway and gut microbiomes, especially of hospitalized preterm and term neonates [24-26]. Multilocus sequence typing (MLST), a nucleotide sequence based technology, is an advanced tool of investigating bacterial evolution and epidemiological pattern [8,25,27-30]. Current data demonstrate that *S. epidermidis* strains isolated from hospitalized neonates are not widely diverse among various regions, but are represented by a limited number of clones [8,25,28,30]. The hands of health-care personnel are the major sources of *S. epidermidis* transmission among neonates [8,25,28,30].

## Incidence of *S. epidermidis* sepsis and neonatal inflammatory diseases

The emergence of *S. epidermidis* as the most common pathogen of neonatal sepsis is closely associated with paradigm shifts in perinatal and neonatal medicine. While the universal prenatal screening and treatment protocol for Group B *Streptococcus* (GBS) have contributed to a significant reduction in early-onset sepsis (EOS), the incidence of nosocomial late-onset sepsis (LOS) has significantly increased [3, 31]. LOS is most frequently defined as sepsis occurring after 72 h of age, and has been well recognized to be associated with prematurity, invasive interventions like intravascular catheterization, failure in early enteral feeding, prolonged antibiotic treatment and hospitalization, as well as underlying respiratory and cardiovascular diseases [3]. The omnipresence of *S. epidermidis* on human skin and host susceptibility may allow *S. epidermidis* to easily invade into the bloodstream through indwelling catheters [3,4]. Despite intensive efforts to reduce nosocomial infections, an average of one third of VLBW infants would still have at least one episode of LOS [9, 32-43]. The incidence of LOS increases up to 50% in very immature preterm infants with a gestational age (GA) ≤ 24 weeks, compared to 24–30% and 8–19% in neonates with GA 25–26 weeks and 27–28 weeks, respectively [2]. Over the last 5–10 years, the proportion of LOS caused by *S. epidermidis* in most countries remained rather unchanged at around 50% (Table 1) [9, 32-43].

**Table 1.** The proportion of culture-proven late-onset sepsis due to coagulase-negative Staphylococci, predominantly *Staphylococcus epidermidis*, among different geographical areas.

Region	Birth year	Design	Population	No. of LOS cases	% of LOS due to CONS	Ref
Developed countries						
Netherland	2008-2014	SC	GA < 32 wk	86	77.9	Claessens et al.,^32^ 2017
			and/or VLBW			
Canada	2015	MC	GA < 32 wk	429	58.9	CNN,^33^ 2016
France	1997	MC	GA 22-32 wk	816	46.0	Mitha et al.,^34^ 2013
Australia	2005-2016	SC	Neonates in	146	39.8	Gowda et al.,^35^ 2017
			NICU			
South Korea	2013-2014	MC	VLBW	442	38.3	Lee et al.,^9^ 2015
USA	2000-2011	MC	ELBW	2000-2005: 2083	50.0	Greenberg et al.,^36^ 2017
				2006-2011: 1728	57.0	
UK	2002-2011	SC	VLBW	2002-2007: 379	26.7	Davis et al.^37^ 2015
				2008-2011: 378	14.1	
Germany	2012-2014	MC	VLBW	133	13.0	Tröger et al.,^38^ 2016
Developing countries						
Poland	2009-2011	MC	VLBW	304	62.5	Wójkowska-Mach et al.,^39^ 2014
Ghana	2010-2013	SC	Neonates in	1039	52.8	Labi et al.,^40^ 2016
			NICU			
China	1990-2014	SC	Neonates in	587	49.8	Lu et al.,^41^ 2016
			NICU			
Turkey	2003-2010	SC	GA < 37wk	86	40.0	Ozkan et al.,^42^ 2014
Malaysia	2010	MC	VLBW	562	25.6	Boo et al.,^43^ 2016

CONS: coagulase negative Staphylococci; LOS: late-onset sepsis; MC: multiple centers; NICU: neonatal intensive care unit; SC: single center; VLBW: very low birth weight.

Accumulating data demonstrate that *S. epidermidis* sepsis confers an increased risk of adverse short-term and long-term neonatal outcome, especially in very immature preterm infants. Although prematurity, genetic predisposition, nutritional deficits and hemodynamic instability may also contribute to BPD, WMI, NEC and ROP [17-20], *S. epidermidis* sepsis has been demonstrated to be an independent risk factor of all these neonatal morbidities [10-16]. Neonates with *S. epidermidis* sepsis were demonstrated to have a significantly higher risk of BPD compared to neonates without sepsis. The relative risk (RR) for BPD varied from 2.6 (95% CI: 1.5-4.6) to 9.40 (95% CI: 3.83-23.08) among different studies [12,16]. In a recent meta-analysis, the odds ratios (ORs) for neurodevelopmental impairment (NDI) and cerebral palsy were found to be 1.31 (95% CI: 1.09-1.57) and 1.7 (95% CI: 1.02-2.87), respectively, in neonates with *S. epidermidis* sepsis compared to non-septic controls [10]. Additionally, white and grey matter abnormality was demonstrated in neonates with *S. epidermidis* sepsis by means of magnetic resonance imaging (MRI) [14]. It has been shown that the incidence of NEC and severe ROP ≥ stage 3 in extremely preterm infants with *S. epidermidis* sepsis was 21.9% and 37.1%, respectively, nearly 2 times higher than non-sepsis controls [13]. Apart from these, there might be an enhanced risk of growth retardation in neonates with *S. epidermidis* sepsis compared to uninfected neonates (95% CI of OR: 1.1-1.7) [15]. Notably, the sepsis-associated risk of BPD, NDI and growth retardation does not seem to differ between *S. epidermidis* and other pathogens, namely coagulase-positive staphylococci, gram-negative bacteria and fungi [11,12,14,15]. This suggests that a common pathway of inflammation initially triggered by diverse pathogens may contribute to neonatal long-term adverse outcomes. In general, these results challenge the conventional conception of *S. epidermidis*is being a non-virulent microorganism, and underscored a significant role of *S. epidermdis* sepsis in the pathogenesis of neonatal inflammatory diseases.

## Putative virulent factors of *S. epidermidis* involved in neonatal sepsis

Abundant research has demonstrated that most of the pathogenic determinants of *S. epidermidis* have their original roles in a commensal life, including outcompeting pathogens for physiological niches in the host, inhibition of other harmful microorganisms and priming of innate immune responses [44-46]. Factors associated with nosocomial environmental, such as skin-breaching interventions, and individual susceptibility may contribute to the role switch of *S. epidermidis* from a commensal to a nosocomial pathogen [36, 47]. According to investigations carried out in the last two decades, *S. epidermids* sequence type 2 (ST2) appears to be the most widespread nosocomial clone in neonatal ward, and may account for the majority of cases of neonatal sepsis [8, 25,28,30]. ST2 is characterized by biofilm forming capacity, antibiotic resistance and a highly flexible genetic background [27-30]. This sends an alarming signal that pathogenicity of *S. epidermidis* may evolve with the progress in medical technology and changes in patient demographics, and indicates the necessity of reevaluating the role of *S. epidermidis* in nosocomial infections. In the following, putative virulent factors implicated in *S. epidermidis* sepsis will be reviewed, with a special focus on their roles in bacteria-host interaction. For a more comprehensive understanding of *S. epidermidis* pathogenic factors, readers are referred to a review elsewhere (see [ref. 48).

## Biofilms

Bacterial biofilm is a highly organized society of bacteria embedded in self-produced extracellular polymeric matrix [49]. Its development follows a three-stage process comprising attachment, accumulation and detachment [49]. The attachment of bacteria on a biotic surface is mainly mediated via microbial surface components recognizing adhesive matrix molecules (MSCRAMMs) [50]. Cell surface hydrophobicity is another important mediating factor in bacterial interaction with abiotic surfaces [51]. Subsequently, both bacterial proliferation and the secretion of various extracellular polymers contribute to the formation of small aggregates, which then mature into biofilm with a three-dimensional structure and enhanced resistance against antibiotics and immune cells [49,52,53]. *S. epidermidis* has been shown to form biofilms on medical devices within 24 h of invasion [51]. As maturation proceeds, bacteria may disperse from the biofilm and colonize new infection sites [52]. Although chronic and indolent by nature, biofilm formation should not be excluded from the pathogenic mechanisms of neonatal sepsis, which is otherwise considered to be an acute disease. Bacteremia and the subsequent inflammation are considered to partly originate from the dispersal of bacteria from biofilms on indwelling medical devices [48,54]. Moreover, the persistence of biofilms may provide a base for a continuous seeding of bacteria into the bloodstream [48,54]. This may largely explain why persistent *S. epidermidis* sepsis is not rare among very immature preterm neonates during hospitalization [16,55].

## Extracellular polymers and surface components of *S. epidermidis*

Polysaccharide intercellular adhesin (PIA), also named poly-N-acetylglucosamine in terms of its basic structure, is the best characterized *S. epidermidis* extracellular polymer and a major constituent of *S. epidermidis* biofilms [56]. The *icaADBC* operon regulates the synthesis of PIA, and a small insertion sequence (IS) element IS*256* may switch biofilm phenotype through reversible transposition into the *icaADBC* locus [27,30]. *S. epidermidis* strains positive for *ica* and IS*256* are preferentially found in nosocomial epidemic clones like ST2, emphasizing the significance of PIA and genetic flexibility in *S. epidermidis* pathogenicity [27-30]. PIA has been demonstrated to increase the amount of C5a in human serum, reduce the sensitivity of *S. epidermidis* to human antimicrobial peptides (AMPs) such as LL-37 and β-defensin 3, inhibit the phagocytosis of human neutrophils and macrophages, and enhance bacterial survival in phagosomes [57-59]. Compared to PIA-negative *S. epidermidis* strains, PIA-positive strains seem to induce lower levels of pro-inflammatory cytokines including tumor necrosis factor-α (TNF-α), interleukin (IL)-1β, IL-6, IL-12p40 and IL-12p70, but a higher level of IL-8, a potent neutrophil recruiting factor [57-59]. In contrast to PIA, poly-γ-DL-glutamic acid (PGA) is produced by strains both of clinical and commensal origins, and may have minimal impact on *S. epidermidis* biofilm formation [60]. Nonetheless, *S. epidermidis* strains incapable of producing PGA were more likely to be phagocytosed by human neutrophils and killed by LL-37 and β-defensin 3 than PGA-positive strains [60]. Recently, a novel lipopeptide (LP01) produced by *S. epidermdis* has been identified to be a Toll-like receptor 2 (TLR2) agonist, and may trigger the expression of β-defensins [61]. In addition, *S. epidermidis* surface components lipoteichoic acid (LTA) and peptidoglycan (PGN) may increase the production of TNF-α, IL-1β, IL-6 and nitric oxide (NO) in murine macrophages [62]. The overall pro-inflammatory effect of *S. epidermidis*, however, was demonstrated to be weaker than that provoked by *E. coli* [62]. These results underscore that the interaction between *S. epidermidis* and the host immunity originally relies on immune evasion, rather than aggressive invasion [54].

## Toxins, exoenzymes and other virulent factors

For pathogenic microorganisms, toxins are considered to be major virulence factors. In contrast to the vast repertoire of toxins produced by other virulent pathogens, such as *S. aureus* and *E. coli*, toxin production in *S. epidermidis* is predominantly restricted to phenol-soluble modulins (PSMs) [63]. PSMs comprise a group of amphipathic, α-helical peptides which are widely produced by staphylococcal species, and can be categorized into shorter α-type peptides (20-25 amino acids) and longer β-type peptides (43-45 amino acids) [63]. PSMs of *S. epidermidis* include PSMα, PSMδ, PSMε, δ-toxin and PSM-mec, which all belong to α-type peptides, as well as PSMβ1 and PSMβ2 [63]. PSMs may contribute to *S. epidermidis* pathogenicity in several ways. First, PSMs have surfactant-like properties, which may shape biofilm into a mature three-dimensional structure and facilitate bacterial dispersal [52]. Second, some α-type PSMs of *S. epidermidis*, especially PSMδ, are potent cytolytic agents of human neutrophils at micromolar concentrations [64]. However, the composition of α-type PSMs in relation to non-cytolytic β-type PSMs in *S. epidermidis* was found to be lower than in *S. aureus* [64]. Additionally, the production profile of PSMs appears to be highly strain-dependent, and some clinical invasive strains may have an overall enhanced production of PSMs [64,65]. Third, some PSMs produced by *S. epidermidis*, such as PSMδ, PSMε, PSM-mec and PSMβ1, are powerful pro-inflammatory agents which may stimulate IL-8 release even at nanomolar concentrations [64,65]. Fourth, certain PSM of *S. epidermidis* was demonstrated to be enteropathic, and may contribute to the development of NEC in preterm infants [66]. The production of most PSMs is regulated by *agr* quorum-sensing system, a core-genome regulator which also influences the expression of MSCRAMMs and exoenzymes [67]. In contrast, PSM-mec is encoded by *psm-mec* gene locus, located within the mobile genetic element staphylococcal chromosome cassette *mec* (*SCCmec*), which mediates methicillin resistance in *S. epidermidis* [27,65]. The emergence of *psm-mec* by combining two pathogenic elements in one genetic exchange event is a good example of *S. epidermidis* genetic flexibility [65]. In a mouse model of neonatal sepsis, PSM-mec significantly increased the expression of IL-1β, TNF-α and the mouse IL-8 homologue CXCL1, leading to increased mortality [68]. This recent study by Qin et al [68]. was the very first to suggest an important role of PSM-mec in the pathogenesis of *S. epidermidis* sepsis.

Other virulent factors of *S. epidermidis*, such as exoenzymes, have been demonstrated to be mainly involved in *S. epidermidis* immune evasion. Protease SepA may contribute to the resistance of *S. epidermidis* against human AMPs and neutrophils [64]. Endopeptidase Esp was found to degrade host fibronectin and complement factors [69]. Furthermore, *S. epidermidis* can produce lipases GehC and GehD to inactivate host-derived fatty acids [70]. As an alternative strategy to circumvent host immunity, *S. epidermidis* may employ Aps AMP sensor/resistance regulator to repel AMPs and enhance its survival after being phagocytosed [64].

Taken together, the generally passive nature of *S. epidermidis* pathogenicity is consistent with its commensal origin. However, the nosocomial environment and individual state of host immunity may drive *S. epidermidis* to transform into a nosocomial pathogen by adjusting its expression of pathogenic components. *S. epidermidis*-induced neonatal inflammation may reflect the net effect of a consortium of intricately orchestrated virulent factors interacting with the host immune system.

## Neonatal innate immune response to *S. epidermidis*

As *S. epidermidis* sepsis is acute by nature, neonatal host immune response to *S. epidermidis* is considered to be mainly mediated by innate immunity [71,72]. Accumulating data have demonstrated that neonatal innate immunity may be partially deficient in quantity and/or quality, with a distinctive regulation pattern of inflammatory response [72,73]. Until now, neonatal host response to *S. epidermidis* sepsis has not been fully elucidated.

## Phagocytosis and intracellular killing of *S. epidermidis*

Compared to term neonates and adults, preterm neonates seem to be deficient in serum levels of complement and AMPs, as well as in the proportion of phagocytic neutrophils and monocytes [74-76]. However, both phagocytosis and intracellular killing of *S. epidermidis* were demonstrated to be similar between preterm and term neonatal monocytes [76]. Upon stimulation with *S. epidermidis*, neutrophils of preterm infants may exert a lower oxidative burst activity than those of term neonates [77]. This indicates that oxygen radical-independent pathways might contribute to the uncompromised capacity of preterm neonatal neutrophils to kill *S. epidermidis* intracellularly. Future studies are warranted to confirm this assumption. Notably, neutrophil oxidative burst responses to *S. epidermidis* seem to vary among strains [77]. Sepsis-associated strains tend to induce an enhanced oxidative burst activity compared to non-sepsis strains [77]. In order to accurately interpret bacteria-host interactions, strain phenotype of *S. epidermidis* should be characterized to identify clinical strains which are more virulent than others. On account of the often unspecific information on strain characteristics in current studies, the phagocytosis and intracellular killing capacity of *S. epidermidis* by neonatal phagocytes remain to be elucidated.

## S. *epidermidis*-driven cytokine and chemokine responses

Data concerning *S. epidermidis*-induced inflammatory mediators in neonates are predominantly based on *in vitro* experiments (Table 2). *S. epidermidis* was demonstrated to induce the release of pro-inflammatory cytokines such as TNF-α, IL-1β, IL-6, IL-8 and IL-12 [78-83]. This induction of pro-inflammatory cytokines appears to be dependent on time, bacterial inoculum and strain phenotype. The levels of TNF-α, IL-1β, IL-6 and IL-8 may show a rapid rise by several folds within the first 4 h upon *S. epidermidis* exposure [79]. Intracytoplasmic IL-6 production was demonstrated to be nearly 4 times higher at a multiplicity of infection value of 10:1 (colony forming unit: white blood cell) than at 1:1 [81]. Furthermore, *S. epidermidis* strains positive for *icaADBC* seem to induce a higher level of IL-8 while inhibiting the release of IL-6 as compared to *icaADBC* negative strains [81,83]. This may be associated with the pathogenicity of clinically invasive *S. epidermidis* clones.

**Table 2.** *In vitro* and *in vivo* studies reporting neonatal host inflammatory responses to *Staphylococcus epidermidis*.

Study	Bacterial strain	Preparation	Patient population	Intervention	Results
			Cell or animal type		
*In vitro* experiments					
Strunk et al.,^78^ 2012	SE 1457	Heat-killed	GA<30wk, 31-33 wk, 37-41 wk	6 h	IL-1β, IL-6, IL-8, TNF-α↑with GA
		10^8^cfu/ml	Cord & adult peripheral MNCs		Adult level TLR2 expression & phagocytosis
Mohamed et al.,^79^ 2007	SE PT 9657	Heat-killed	Healthy term infants	4 h	IL-1β, IL-6, IL-8, TNF-α↑with time
		10^3^cfu/ml	Cord & adult peripheral WBC		Pro-inflammatory effect: SE <*E.coli* & GBS
Tatad et al.,^80^ 2008	Clinical isolate	Heat-killed	Healthy term & preterm infants	18 h	IL-6, IL-8, IL-10, IL-12↑ similar to adult
		10^4^cfu/ml	Cord & adult peripheral WBC		Inflammatory response: preterm > term
Härtel et al.,^81^ 2007	ATCC 12228	Live bacteria	Term & preterm infants	24 h	IL-6, TNF-α↑with GA
	Strain 94B080	1cfu:1 WBC &	Neonatal peripheral WBC		Pro-inflammatory effect: BF^+^ strain<BF^-^ strain
	Strain 94B575	10 cfu:1 WBC			IL-10, TGF-β expression independent of GA
Peoples et al.,^82^ 2009	Clinical isolate	Heat-killed	Healthy term & preterm infants	18 h	IL-6, IL-10↑> adult
		10^4^ cfu/ml	Cord & adult peripheral WBC		IL-8, IL-12, INF-NK, INF-T↑ similar to adult
					Inflammatory response: preterm=term
Haase et al.,^83^ 2011	ATCC 12228	Live bacteria	Healthy term & preterm infants	1 h	TNF-α, IL-6, IL-8↑
	Strain 94B080	1cfu:1 WBC	Cord WBC		Pro-inflammatory effect: BF^+^ strain>BF^-^ strain
	Strain 94B575				
Björkqvist et al.,^77^ 2004	Clinical isolate	Live bacteria	Healthy term & preterm infants	30 min	Oxidative burst intensity: preterm < term
		10 cfu:1 PMNL	Neonatal peripheral PMNLs		SE similar to GBS
Ivarsson et al.,^104^ 2013	Clinical isolate	Live bacteria	Vascular endothelial cells	18 h	IL-8, ICAM-1 ↑
		10^4^cfu/ml	Small airway epithelial cells		Pro-inflammatory effect: SE > SA
Hussain et al.,^105^ 2013	Strain 94B080	Live bacteria	Bronchial epithelial cells	36 h	TNF-α, IL-6, IL-8 and iNOS↑ with time
		10^4^cfu/ml			Expression of ENaC and CFTR↑
*In vivo* experiments					
Kronforst et al.,^87^ 2012	SE 1457	Live bacteria	C57BL/6 WT mice	48 h	At 2 h:
		up to 2×10^9^ cfu/ml	< 24 h old		IL-1β, IL-6, TNF-α, TLR2↑ with inoculum
					At 24 h & 48 h:
					Inoculum-dependent neonatal weight loss
Bi et al.,^86^ 2015	SE 1457	Live bacteria	C57BL/6J WT mice	72 h	At 6 h:
		1×10^8^cfu/ml	< 24 h old		IL-6, CCL2, CXCL1, IL-12↑ in blood & brain
					Caspase-3 & TLR2 mRNA ↑ in the brain
					At 24 h:
					PMNLs↑ in blood & cerebral spinal fluid
					White and grey matter impairment

BF: biofilm; cfu: colony forming unit; CFTR: cystic fibrosis transmembrane conductance regulator; ENaC: Epithelial Na+ channel; GA: gestational age; GBS: group B *Streptococcus*; ICAM-1: intercellular adhesion molecular-1; IL: interleukin; INF: interferon; MNCs: mononuclear cells; NK: natural killer; PMNLs: polymorphonuclear leucocytes; SA: *Staphylococcus aureus*; SE: *Staphylococcus epidermidis*; TLR: Toll-like receptor; TGF: transforming growth factor; TNF: tumor necrosis factor; WBC: whole blood cell; WT: wild type.

Discussions are ongoing regarding the pro-inflammatory capacity of *S. epidermidis* compared to other pathogens. It has been demonstrated that levels of TNF-α, IL-1β, IL-6 and IL-8 induced by *S. epidermidis* at 2 h and 4 h upon stimulation were several times lower than those induced by *E. coli* and GBS [79]. In contrast, another study showed that the release of IL-6, IL-8 and IL-12 at 18 h of infection did not differ among *S. epidermidis*, *E. coli* and GBS [82]. Discrepant results also exist concerning *S. epidermidis*-induced pro-inflammatory response in relation to neonatal maturity. Some studies reported a GA-dependent increase of *S. epidermidis*-induced TNF-α, IL-1β, IL-6 and IL-8 [78,81], while others demonstrating similar concentrations of IL-6, IL-8 and IL-12 in preterm and term neonates [80,82]. Anti-inflammatory IL-10 was consistently shown to be produced in a GA-independent manner [80-82]. The net effect of counterbalancing pro- and anti-inflammatory cytokines was mostly unexplored in *in vitro* studies of *S. epidermidis*, with only one study demonstrating a higher production of pro-inflammatory factors than anti-inflammatory factors [80]. It should be noted that perinatal factors such as *in utero* exposure to infection/inflammation, hyperoxia/hypoxia, and temperature instability are likely to affect the signaling of immune cells [84]. These aspects may, to some extent, explain the inconsistency among studies. Furthermore, most *in vitro* studies used heat-killed *S. epidermidis*, which has been demonstrated to mount a different pattern of cytokine production compared to live bacteria [85]. Other confounding factors may include TLR2 gene polymorphism [84], *S. epidermidis* strain phenotypes [83], and experimental methods.

Performance of *in vitro* studies using cell lines may not accurately reflect the bacteria-host interaction. By comparison, *in vivo* animal studies have the advantage to explore the release pattern of inflammatory cytokines and the impact of inflammatory response on host. Currently, there is a dearth of studies providing such information [86,87]. Kronforst et al. and Bi et al. are the first to establish a mouse model of neonatal *S. epidermidis* sepsis [86,87]. At 2–6 h post infection, *S. epidermidis* nosocomial strain 1457 was demonstrated to induce a rapid production of cytokines, such as TNF-α, IL-1β, IL-6, IL-12p70 and IL-10, as well as chemoattractant protein-1 (MCP-1), granulocyte-colony stimulating factor (G-CSF), CCL2 and CXCL1 [86,87]. Moreover, the production profile of cytokines was found to increase with bacterial inoculum, and to shift towards pro-inflammation as IL-10 was down-regulated compared to pro-inflammatory cytokines [87]. As a result, newborn mice suffered significant weight loss at 24 h and 48 h post infection [87], followed by white and grey matter injury observed at the 14^th^ day after birth [86].

## Signaling pathways involved in the host immune response to *S. epidermidis* sepsis

The host immune response against *S. epidermidis* is found to be largely mediated by TLR2 [88]. The pathogenic factors PIA, LTA, PGN, lipopeptides and PSMs of *S. epidermidis* are well-recognized ligands for TLR2 [61,89-91]. Recently, formyl peptide receptor 2 (FPR2) and nucleotide oligomerization domain (NOD)-like receptor NOD2 have been shown to be additional receptors for PSMs and PGN, respectively [92-94]. Activation of TLR2, FPR2 and NOD2 uniformly culminates in nuclear factor-κB (NF-κB) transcription and the synthesis of inflammatory mediators [84,95,96]. The expression of TLR2 and downstream signaling molecules appears to be similar between neonates of different GA and adults [97-98]. The same pattern of expression has been demonstrated for NOD2 [99]. Moreover, TLR2 expression may be up-regulated in neonates with sepsis, supporting the hypothesis of dysregulated neonatal inflammatory response in favor of hyper-inflammation [100,101]. As for FPR2, we found no data concerning its expression in neonates. A study using a mouse model of neonatal sepsis indicated that at high bacterial concentrations, FPR2 and NOD2 may be more involved in the recognition of *S. epidermidis* than TLR2 [86].

In general, in the scenario of established sepsis caused by clinically invasive *S. epidermidis* strains, neonatal immune response to *S. epidermidis* appears to skew towards hyper-inflammation, potentially leading to tissue damages and long-term morbidity. However, more studies are warranted to verify this pattern of *S. epidermidis*-induced inflammatory response in neonates.

## S. *epidermidis*-induced inflammation and neonatal diseases

**Figure 1.**
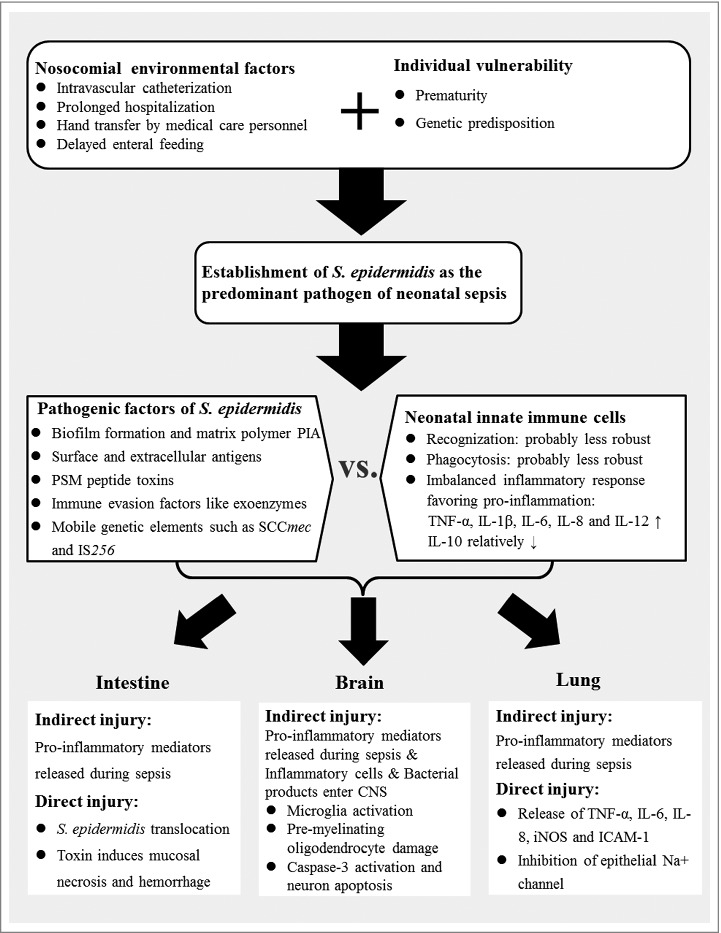
Putative mechanisms of *Staphylococcus epidermidis*-induced neonatal inflammatory response and consecutive organ injuries. PIA: polysaccharide intercellular adhesin; PSMs: phenol-soluble modulins; SCC*mec*: staphylococcal chromosome cassette *mec*; IS: insertion sequence; TNF-α: tumor necrosis factor-α; IL: interleukin; CNS: central nervous system; iNOS: inducible nitric oxide synthase; ICAM-1: intercellular adhesion molecule-1.

The role of *S. epidermidis* sepsis in neonatal inflammatory diseases, such as BPD, WMI, NEC and ROP, has just begun to be unraveled. So far, data concerning the mechanisms of *S. epidermidis* sepsis-driven ophthalmologic injury are lacking. In regard of other inflammatory disorders, exposure to pro-inflammatory factors such as TNF-α, IL-1β, IL-6 and IL-8, in combination with a deficiency of anti-inflammatory factor IL-10, has been demonstrated to cause lung tissue damages as well as the breakdown of intestinal epithelial integrity and blood-brain barrier [17,18,102,103]. This may confer indirect evidence of inflammation-induced organ injuries associated with *S. epidermidis* sepsis (Figure 1). Moreover, there is emerging evidence of *S. epidermidis* directly interacting with neonatal tissues, which will be elaborated in this part of the review.

## Bronchopulmonary dysplasia

BPD is characterized by dysregulated alveolarization and impaired angiogenesis, and has become the most common complication of preterm births [17]. The incidence of BPD is estimated to be 50% in extremely preterm neonates [2]. So far, direct evidence linking *S. epidermidis* infection and lung injury is scarce. One *in vitro* study has shown that *S. epidermidis* may stimulate higher levels of IL-8 and intercellular adhesion molecule-1 (ICAM-1) than *S. aureus* in vascular endothelial cells and small airway epithelial cells, whereas IL-10 was undetected [104]. As IL-8 and ICAM-1 are strong chemoattractants, the authors speculated that *S. epidermidis* sepsis might contribute to the pathogenesis of BPD by generating a persistent recruitment of inflammatory cells into lung tissues [104]. In another *in vitro* study using bronchial epithelial cells, *S. epidermidis* was demonstrated to increase the production of IL-6, IL-8, TNF-α and inducible nitric oxide synthase (iNOS) in a time-dependent manner [105]. Furthermore, activities of epithelial Na+ channel (ENaC) and cystic fibrosis transmembrane conductance regulator (CFTR), both being critical in the regulation of lung liquid homeostasis, were enhanced upon stimulation of *S. epidermidis* [105]. The role of *S. epidermidis* infection in the pathogenesis of BPD remains controversial. Some suggested that *S. epidermidis* might colonize in the lung and persistently produce a low-grade inflammation to cause BPD [104]. Another assumption is based on the finding that infection-induced inflammatory mediators, such as neutrophils and cytokines, may not be detected several days after the onset of infection [106]. Therefore, a pro-inflammatory response initiated by *S. epidermidis* sepsis may act as a contributing hit. Subsequently, sustained activation of the lung inflammatory cascade in the context of prematurity and dysregulated neonatal immunity may contribute to the pathogenesis of BPD.

## White matter injury

The most common form of preterm brain injury is white matter injury (WMI), characterized by focal and diffuse abnormalities in cerebral white matter with loss of pre-myelinating oligodendrocytes, inhibition of neuronal precursor cell proliferation, and excessive activation of microglia [18,107,108]. Neonatal brain injury following bacteremia/sepsis may be due to the penetration of bacteria into central nervous system (CNS), as in the case of meningitis caused by *E. coli* and GBS [109]. In contrast, *S. epidermidis* is less virulent and invasive, and barely causes meningitis [4]. However, *S. epidermidis*-induced systemic cytokines, inflammatory cells, and bacterial products, such as toxins and surface components, may enter the CNS through a disrupted blood-brain barrier [18,86]. The study conducted by Bi et al. is the first to provide evidence of a causal relationship between *S. epidermidis* sepsis and neonatal brain injury [86]. In this model, culture results from the brain as well as the cerebrospinal fluid (CSF) were persistently negative of *S. epidermidis* [86]. However, the white blood cell count in CSF and CCL2 in the brain increased upon *S. epidermidis* sepsis at 2 h [86]. At 6 h post infection, the expression of caspase-3, which may activate microglia and trigger brain apoptosis, was enhanced in brain tissues [86]. Of note, 14 days postnatally, decreased volumes of white and grey matter as well as disruption of myelination at the cortical region were detected [86]. In light of the above, *S. epidermidis* may directly trigger an imbalanced systemic pro-inflammatory reaction, and may also indirectly stimulate resident cerebral immune cells to release cerebral inflammatory mediators, leading to the damage of pre-myelinating oligodendrocytes and neurons [18,86]. These effects may act in concert to result in white and grey matter abnormalities later in life [18,86].

## Necrotizing enterocolitis

NEC is the most common gastrointestinal emergency among premature infants [19,110]. Characterized by intestinal inflammation that can progress to necrosis and perforation, NEC often confers life-long sequelae in survivors [19,110]. Although bacteria, including *S. epidermidis*, have been clearly demonstrated to be involved in the pathogenesis of NEC, there is not a single species that has been found to be the determinant pathogen [19]. Instead, microbial dysbiosis and disrupted gut intestinal barrier are speculated to be major underlying mechanisms [111]. The expression of TLR2 on intestinal epithelia seems to be up-regulated during NEC [19], which may further enhance the susceptibility of the immature gut to pathogens. Besides indirect evidence linking *S. epidermidis* sepsis and gut injury, there is also direct evidence indicating that *S. epidermidis* was capable to translocate into the gut cavity through injured intestine [112]. Results from very recent studies corroborated this finding and demonstrated that the causative agent of LOS, including *S. epidermidis*, may correspond to the abundant bacterial genera in gut microbiome at the diagnosis of LOS [24,25]. The gut-colonizing *S. epidermidis* seems to be characterized by the carriage of pathogenic factors, such as *icaA*, IS*256*, SCC*mec* and toxins [25,66]. Toxins of *S. epidermidis* were found to induce mucosal necrosis and hemorrhage in the bowel [66]. Taken together, *S. epidermidis* may be implicated in the pathogenesis of NEC, probably not as a causative pathogen but as a conspirator. The contribution of *S. epidermidis* sepsis to NEC remains to be elucidated.

## Retinopathy of prematurity

As a retinal vascular disorder most commonly seen in preterm neonates, ROP is the major cause of visual impairment or blindness in children [113]. The development of ROP comprises an initial phase of inhibited retinal vessel growth followed by a second phase of abnormal vasoproliferation [114]. Although prematurity and oxygen exposure are central in the etiology of ROP, neonatal inflammation and infection may have a deleterious impact on retinal angiogenesis, aggravating the risk of ROP [114,115]. Pro-inflammatory cytokines, such as TNF-α and IL-6, as well as vascular endothelial growth factor (VEGF) and insulin-like growth factor 1 (IGF-1) are associated with dysregulated vascularization [20,114]. *S. epidermidis* is one of the prevalent pathogens of endophthalmitis in adults, indicating its potential to cause ocular inflammatory injury [116]. However, direct evidence linking *S. epidermidis* to ROP has not been found yet. Based on current knowledge, *S. epidermidis* is not likely to play a key role in the development of ROP following *S. epidermidis* sepsis, which predominantly occurs in very immature preterm infants who are also at an enhanced risk of oxygen exposure [2]. Conversely, *S. epidermidis* sepsis-driven inflammation may, through indirect mechanisms, exacerbate the dysregulated process of angiogenesis leading to ROP.

## Future perspectives

Despite being the most frequently isolated pathogen of neonatal sepsis, the significance of *S. epidermidis* in neonatal diseases is recognized to a lesser extent compared to other virulent pathogens, such as *S. aureus* and *E. coli*. However, due to the enhanced awareness that *S. epidermidis* may considerably contribute to neonatal short-term and long-term morbidity via inflammation-induced organ injury, epidemiological and experimental studies are warranted in the future.

How can current knowledge be implicated in the clinical management of neonatal sepsis? Close surveillance of the distribution of epidemic clones and characterization of pathogenic elements of *S. epidermidis* strains may improve infection control. Moreover, the prevention of preterm births as well as the reduction of invasive procedures and strict hand hygiene will still constitute the cornerstone of preventive measures against *S. epidermidis* sepsis [3]. For patients with established sepsis, our armamentarium of anti-sepsis strategies is still highly restricted to antibiotics. Given the potential role of sepsis-induced inflammation in the pathogenesis of neonatal morbidity, new strategies of immunomodulation aiming at restoring the balance of neonatal inflammatory response might add to current sepsis treatment protocols [21].
